# Molecular Profiling of Kenyan Acute Myeloid Leukemia Patients

**DOI:** 10.3389/fgene.2022.843705

**Published:** 2022-06-28

**Authors:** Mercy Gatua, Mohsen Navari, Matilda Ong’ondi, Noel Onyango, Serah Kaggia, Emily Rogena, Giuseppe Visani, Nicholas A. Abinya, Pier Paolo Piccaluga

**Affiliations:** ^1^ Biobank of Research, IRCCS S. Orsola-Malpighi Academic Hospital, Department of Experimental, Diagnostic and Specialty Medicine, Institute of Hematology and Medical Oncology “L. and A. Seràgnoli”, University of Bologna School of Medicine, Bologna, Italy; ^2^ Kenyatta National Hospital, Nairobi, Kenya; ^3^ Department of Medical Biotechnology, School of Paramedical Sciences, Torbat Heydariyeh University of Medical Sciences, Torbat Heydariyeh, Iran; ^4^ Bioinformatics Research Group, Mashhad University of Medical Sciences, Mashhad, Iran; ^5^ Research Center of Advanced Technologies in Medicine, Torbat Heydariyeh University of Medical Sciences, Torbat Heydariyeh, Iran; ^6^ Nairobi Hospital, University of Nairobi, Nairobi, Kenya; ^7^ Department of Pathology, School of Medicine, Jomo Kenyatta University of Agriculture and Technology, Juja, Kenya; ^8^ Hematology and Stem Cell Transplantation, AORMN, Pesaro, Italy; ^9^ Istituto Euro-Mediterraneo di Scienza e Tecnologia (IEMEST), Palermo, Italy; ^10^ Nanchang University, Nanchang, China

**Keywords:** acute myeloid leukemia, next generation sequencing, illumina, myeloid panel, ELN, cytogenetics, targeted therapy

## Abstract

Acute myeloid leukemia (AML) is an infrequent disease, and it is associated with high morbidity and mortality. It harbors a unique configuration of cytogenetic abnormalities and molecular mutations that can be detected using microscopic and molecular methods respectively. These genetic tests are core elements of diagnosis and prognostication in high-income countries. They are routinely incorporated in clinical decision making, allowing for the individualization of therapy. However, these tests are largely inaccessible to most patients in Kenya and therefore no data has been reported on this group of patients. The main purpose of this study is to describe the cytogenetic and molecular abnormalities of acute myeloid leukemia patients seen at the hemato-oncology unit of Kenyatta National Hospital. A cross-sectional descriptive study was carried out over a 3-month period on ten patients with a diagnosis of AML. Social demographics and clinical data were collected through a study proforma. A peripheral blood sample was collected for conventional metaphase G-banding technique and next generation sequencing. Particularly, targeted DNA sequencing (Illumina myeloid panel) and whole exome sequencing (WES) were performed. Cytogenetic analysis failed in 10/10 cases. Targeted sequencing was successfully obtained in 8 cases, whereas WES in 7. Cytogenetic studies yielded no results. There were 20 mutations detected across 10 commonly mutated genes. All patients had at least one clinically relevant mutation. Based on ELN criteria, NGS identified three patients with high-risk mutations, affecting *TP53* (*n* = 2) and *RUNX1* (*n* = 1). One patient was classified as favorable (*PML-RARA*) while 4 were standard risk. However, *WT1* mutations associated with unfavorable prognosis were recorded in additional 2 cases. WES showed concordant results with targeted sequencing while unveiling more mutations that warrant further attention. In conclusion**,** we provide the first molecular profiling study of AML patients in Kenya including application of advanced next generation sequencing technologies, highlighting current limitations of AML diagnostics and treatment while confirming the relevance of NGS in AML characterization.

## Introduction

Acute Myeloid Leukemia (AML) is an infrequent disease (De Kouchkovsky and Abdul-Hay, 2016). Although treatment outcomes continue to improve over time, AML is still a significant cause of mortality (Fiegl, 2016). Despite an improvement in the treatment associated mortality, chemo-resistance and post-transplant disease relapse, account for one of the most challenging aspects of AML management (Döhner et al., 2015). It is a heterogeneous clonal disorder that arises from a malignant myeloid stem cell that has acquired genetic and epigenetic mutations that have accumulated in a stepwise fashion. These acquired genetic alterations cause proliferative and survival advantage with reduced apoptosis leading to a buildup of abnormal, poorly differentiated neoplastic cells in the blood and bone marrow with resultant suppression of the normal hematopoietic process (Meyer and Levine, 2014; Döhner et al., 2015).

In Sub-Saharan Africa, acute leukemia causes high mortality. In a 2012 population-based study, the age-standardized rates in East Africa were 3.8 and 3.4 per 100,000 in men and women respectively (Miranda-Filho et al., 2018). Latest Globocan data estimates that in Kenya, the incidence rates of leukemia are 4.8 and 4.5/100,000 in men and women respectively and is listed among the top ten causes of cancer mortality (World Health Organization, 2020). Despite an increasing disease burden in Sub-Saharan Africa, there is limited infrastructure and finances that deters the use of recommended genetic testing (Gopal et al., 2012; Wanjiku et al., 2018). Cytogenetic and molecular techniques, which are core elements of diagnosis in the developed world, are nonexistent in most Sub-Saharan countries (Gopal et al., 2012). Significant financial challenges do exist in emerging economies, where majority of healthcare costs are personal expenditures with many falling below the poverty line. This occurs even in those emerging countries with highly skilled specialists and state of the art facilities that mirror those in developed countries (Philip et al., 2015).

AML cells harbor a unique configuration of cytogenetic and molecular mutations that involve critical genes that regulate the normal hematopoietic process (Löwenberg and Rowe, 2016). This accounts for the phenotypic heterogeneity of the disease (Lagunas-Rangel et al., 2017). Understanding the pathobiology of AML has provided a framework for risk stratification, development of novel treatment approaches with individualization of therapy as well as detection of post treatment minimal residual disease (Döhner and Gaidzik, 2011; Stein, 2015; Dombret and Gardin, 2016; Stein and Tallman, 2016). The urgent need for therapeutic advancement has come at the backdrop of a dismal 5-year overall survival of 50% and 20% for those below and above 60 years old, respectively, with traditional cytotoxic therapies (Stein and Tallman, 2016). A landmark novel anti-leukemic agent should successfully eradicate the malignant founding clone and its sub-clones, eradicating a potential niche for recurrence (Löwenberg and Rowe, 2016). WHO classification of AML incorporates clinical features, morphological assessment of bone marrow specimens, cytochemical studies, immune-phenotyping, cytogenetic and molecular testing to distinguish distinct biological subgroups with clinical importance (Arber et al., 2016). The major categories included in the 2016 WHO classification include; AML with recurrent genetic abnormalities, AML with myelodysplasia-related changes, Therapy-related myeloid neoplasms, AML Not otherwise specified, Myeloid Sarcoma and Myeloid proliferations related to Down syndrome (Arber et al., 2016).

Cytogenetic abnormalities are analyzed using conventional metaphase G-banding techniques and fluorescence *in situ* hybridization (FISH) whereas molecular mutations are detected using next generation sequencing molecular methods (He et al., 2015; Muhammad Ilyas et al., 2015). These technologies are able to comprehensively identify genetic lesions that are critical in the process of leukamogenesis. These genetic abnormalities are the single most powerful prognostic factors and risk stratifies the patient into Favorable, Intermediate and Adverse risk groups (Döhner et al., 2017). Prognostic classification is critical in the management of AML patients, particularly in respect to establishing those with poor prognostic features who are likely to relapse or have chemo-resistant disease. It’s also important for category-specific treatment (Kumar, 2011). However, the prognostic impact of these genetic groups may change with targeted therapy (Gill et al., 2016).

Data from an ongoing prospective study by Prof N. A. Othieno-Abinya (2013 onwards) shows that about 30 patients are diagnosed with AML annually. The median age at diagnosis is 30 years with a male: female ratio of 1.2:1. AML diagnosis at the hemato-oncology unit of KNH is mainly through morphological assessment of bone marrow specimens with few or none of the patients undergoing karyotyping or molecular assessment.

In this study, we therefore aimed to identify the cytogenetic and molecular abnormalities found in patients’ diagnosed with AML at the adult hemato-oncology unit of KNH and prognosticate them according to the EuropeanLeukemiaNet risk stratification model.

## Methods

### Case Selection

The study included patients diagnosed and treated at the adult hemato-oncology unit of Kenyatta National Hospital, the biggest public referral hospital in Nairobi, Kenya. The molecular analyses were carried on at the Department of Experimental, Diagnostic, and Specialty Medicine, Bologna University Italy.

Institutional consent was obtained from the Department of Clinical Medicine and Therapeutics, University of Nairobi (UON) and Ethics and Research Committee of KNH. Request for shipment of the samples for cytogenetic and molecular tests, was obtained from the Ministry of Health subject to the fulfillment of the requirements of KNH-ERC. Material Transfer Agreement between the two institutions was obtained. All patients were informed of the study and a consent/assent obtained in either English/Kiswahili, the national languages of Kenya. For patients aged below 18 years, a legal representative (parent/guardian) gave the consent.

### Cytogenetic Analysis

Cell culture: The cells were cultured using a protocol for harvesting chromosomes from whole blood. In brief, 0.25mls of fresh whole blood was collected in 10 mls of RPMI media containing L-glutamine (20% fetal bovine serum, 1% Penicillin/streptomycin, 1% fungizone, and 1% PHA) and incubated for 48 h at 37°C with 5% CO_2_. Cell counting prior to harvesting of cells revealed that the final seeding densities for each of the samples were less than 1 × 106/ul below the optimum of 1-3 × 106/ul for adequate metaphases. Harvesting was done according to standard protocol and all 10 samples had no evaluable metaphases (Hastings et al., 2013; Howe et al., 2014).

### Next Generation Sequencing

Total DNA was extracted from 8 samples (MG2, MG4i, MG5, MG5i, MG6, MG6i, MG7, MG8) with QIAamp DNA mini kit Qiagen according to the manufacturer’s procedure (Qiagen, Italy). Qubit was used for DNA quality control assessment (ThermoFisher, Italy).

Thereafter, based on Illumina’s TruSeq DNA Sample Preparation, DNA libraries were pre-enriched, according to manufacturer’s instructions (Illumina, United States). Quant-it PicoGreen dsDNA Assay Kit was eventually used for libraries quantification, according to the manufacturer’s protocol (Invitrogen, Life Technologies, United States).

Using Illumina iSeq2500 (Illumina, San Diego, United States), we sequenced the paired-end libraries (2 × 150 base pair), following the manufacturer’s instructions. On average, about 82.9 million 151 bp PF reads were generated, and the theoretical coverage was c143.2, calculated based on hg19 RefSeq non redundant exome length; the median target coverage at 50× was 87.2% (range, 60.7–87.5%). Details on sequencing statistics are described in Supplementary Table S1.

FastQC V0.10.0 tool (http://www.bioinformatics.babraham.ac.uk/projects/fastqc/) was used for quality control, The reads were mapped to Homo sapiens (UCSC hg19) as reference genome, using Burrows-Wheeler Aligner version 2.12.0, while the targeted regions defined by 11062019_ALLEXONV7-NEW-TXT (Li and Durbin, 2009).

To remove potential PCR duplicates, we used SAMtools command rmdup to detect and collapse multiple mapped reads pairs with identical external coordinates (Li and Durbin, 2009). Mapping quality score recalibration and local realignment around insertions and deletions (InDels) was performed using Genome Analysis Toolkit (GATK—v1.6-23-gf0210b3) (McKenna et al., 2010). Single-nucleotide variants (SNVs) and small insertions and deletions (InDels) were called separately using GATK Unified-Genotyper.

All the mutations detected were filtered using thresholds based on quality, coverage, and strand of the mapped reads and according to variants already present in public databases (Hapmap, dbSNP and 1000genome project [The 1000 Genomes Project 2010].

Targeted DNA sequencing utilized the AmpliSeq for illumina myeloid panel. It’s a targeted panel that investigates 62 genes associated with myeloid cancers (Table 1). Library preparation and sequencing were performed as previously described, according to the manufacturer instructions (AmpliSeq for Illumina Myeloid Panel, 2021). Lacking high quality RNA, the gene expression part of the assay was omitted.

**TABLE 1:** Panel of genes tested.

Hot Spot genes	Full genes	Fusion driver genes
*ABL1*	*ASXL*	*ABL*
*BRAF*	*BCOR*	*BCL2*
*CBL*	*CALR*	*BRAF*
*CSF3R*	*CEPBA*	*ALK*
*DNMT3A*	*ETV6*	*CCND1*
*FLT3*	*EZH2*	*CREBBP*
*GATA2*	*IKZF1*	*EGFR*
*HRAS*	*NF1*	*ETV6*
*GATA2*	*PHF6*	*FGFR2*
*IDH1*	*PRPF8*	*FGFR1*
*IDH2*	*RB1*	*FUS*
*JAK2*	*RUNX1*	*HMGA2*
*KIT*	*SH2B3*	*JAK2*
*K-RAS*	*STAG2*	*KMT2A*
*MPL*	*TET2*	*MECOM*
*MYD88*	*TP53*	*MET*
*NPM1*	*ZRSR2*	*MLLTI0*
*N-RAS*		*MYBL1*
*PTPN11*	*MYH11*
*SETBP1*		*NTRK3*
*SRSF2*	*NUP214*
*U2AF1*		*PDGFRA*
*WT1*	*PDGFRB*
		*RARA*
		*RBM15*
		*RUNX1*
		*TCF3*
		*TFE3*

Raw NGS data are available on request (Prof. Piccaluga).

Clinically relevant lesions were defined according to ESMO, NCCN, FDA, and EMA guidelines, as relevant for prognostic and diagnostic significance, or therapy (Li et al., 2017; Heuser et al., 2020; Pollyea et al., 2021).

## Results

### Patients Characteristics

Fifteen AML patients were treated at Kenyatta National Hospital during the study period. Ten consecutives gave the consent and were enrolled. DNA was successfully collected and analyzed in 8/10 cases. The main clinical characteristics of those 8 patients are summarized in Table 2. Briefly, their mean age was 35 years (13-60); 5/8 were males; mean WBC count was 14.3 × 10^9^/L (0.77–20.92); average Hb level was 6.65 g/dl (3.1–10.8); average PLT count was 26.65 × 10^9^/L (6-53). Only one secondary case was reported, previously affected and treated for aplastic anemia.

**TABLE 2:** Patient characteristics.

Case	Age (years)	Gender	% of Marrow Blasts	WBC (×10^9^/L)	Hb (g/dl)	Platelets (×109/L)	Prior Cytotoxic therapy Or Radiotherapy	Antecedent Hematological Disorder
MG2	19	M	30	20.92	6.8	17	NONE	NONE
MG5i	13	F	62	0.77	7.3	46	NONE	NONE
MG4i	50	F	82	44.8	8.2	20	NONE	NONE
MG5	36	M	40	2.86	5.8	11	NONE	APLASTIC ANEMIA
MG6	34	F	44	2.63	4.3	6	NONE	NONE
MG6i	60	M	75	2.60	3.1	44	NONE	NONE
MG7	53	M	70	36	10.8	53	NONE	NONE
MG8	13	M	60	3.49	6.9	16	NONE	NONE

### NGS Revealed Clinically Relevant Genetic Lesions in All Patients

Cytogenetic analysis technically failed in all instances, probably due to the latency between sample collection and analysis (which requires alive cells). DNA quality was, by contrast assessed and confirmed by Qubit (median green RFU 27190.09; range 9697.21–13,262.96).

**FIGURE 1:**
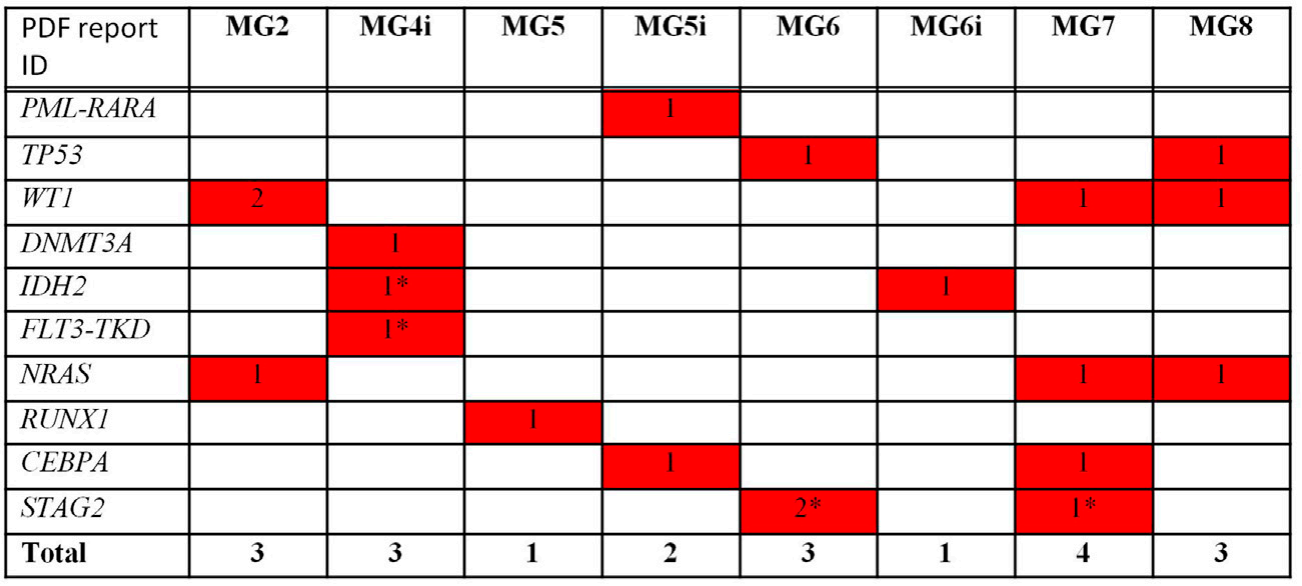
Mutational analysis by NGS—Clinically relevant mutations/Translocations. * not detected at WES.

As far as NGS analysis was concerned, Among the 62 analyzed genes, 20 clinically relevant (as reported by previous publications and common databases) mutations across 10 genes were detected. Of these, 8 were missense mutations, 9 were frame-shift insertions, 1 was a nonsense mutation, and 1 was a fusion gene. The remaining one, affecting FLT3, was a synonymous change. However, it was included among clinically relevant as accepted for accessing clinical trials with FLT3 inhibitors. All patients had at least one clinically relevant mutation with 1/8 cases showing 2 mutations, 4/8 showing 3 mutations each, 2/8 showing 1 mutation each and 1/8 showing 4 (Figure 1).

The most mutated gene was *WT1* (4 mutations in 3 cases), followed by *NRAS* and *STAG2* (3 lesions in 3 cases and 3 lesions in 2 cases, respectively), *TP53*, *IDH2*, and *CEBPA* (2 lesions in 2 cases), and *DNMT3A*, *FLT3*, *RUNX1* (1 lesion). One patient presented with *PML/RARA* rearrangement.

Pairwise mutations to assess for co-mutations could not be undertaken due to the small sample size.

Despite cytogenetics unavailability, NGS results allowed to identify one patient with favorable genetics (*PML/RARA*+), 3 likely to be high risk (*RUNX1* and *TP53*), and 4 standard-risk. Among these, detection of mutations in *IDH2* (N = 2 patients) and *FLT3* (N = 1) allowed to candidate those patients to specific targeted treatments.

### NGS Revealed Clinically Relevant Genetic Lesions of Unknown Significance

In addition to clinically relevant lesions, a series of additional genetic lesions, the significance of which is still undefined, have been identified (Supplementary Table S1).

Briefly, 7/8 patients showed at least one single nucleotide variant (SNV) affecting *SH2B3*, 6/8 *ASXL1* and *TET2* genes, 3/8 *TP53*, 2/8 *ETV6*, *FLT3*, *NF1*, *PTPN11*, while 1/8 *CEBPA*, *GATA2*, *IKZF1*, *KIT*, *PRPF8, RB1,* and *SF3B1.* Despite not being reported as clinically relevant, those disrupting mutations occurred in genes well-known to be associated with myeloid malignancies; therefore, it is conceivable that they might have some pathogenic activity.

### Whole Exome Sequencing Unveiled SNVs Never Reported in AML

Following targeted sequencing of myeloid malignancies associated genes (see above), we sought to perform whole exome sequencing (WES) to further describe the genetic landscape of Kenyan AML patients. The procedure was successful in 7/8 cases.

First, WES confirmed the presence of the clinically relevant lesions observed at targeted sequencing, with the only exceptions of *STAG2* mutations that were not detected in both patients supposed to carry them.

In addition, several SNVs and SNPs were identified. Most of them were not likely associated with cancer and were filtered out. By contrast, a couple of patients showed additional lesions that warrants attention. Patient MG_2 showed an overall quite complex scenario, characterized by several SNVs affecting other 12 genes whose association with cancer (or anti-cancer drug response) is well known. Particularly, *AURKA*, which is associated to colon cancer and was recently found to be over expressed also in hematological malignancies, as well as the drug resistant associated genes *ABCB1, XRCC1* and *CBR3* turned out to be affected. Furthermore, we observed mutations affecting *FGFR4*, *KLC1* and *XRCC3*, associated to colon cancer and melanoma (Supplementary Table S2). Interestingly, *NAT2* might be involved in epigenetic deregulation. Finally, *TP53* turned out to be largely altered, though the identified SNVs are not formally associated with leukemias.

In patient MG_5, known for the previous history of aplastic anemia (AA), we found a *TERC* mutation, known to be associated with AA pathogenesis. By contrast, *TERT*, the other gene associated with AA, presented a synonymous mutation only.

### Clinical Correlates

The limited sample size didn’t allow a proper clinic-pathological correlation. Furthermore, it should be noted that 3/8 patients died before any treatment, including the young acute promyelocytic leukemia patient. Among the remaining 5 patients, 3 died from infection during induction (day 10, day 15 and day 30, respectively), 1 was resistant to induction and died during supportive treatment shortly after, and 1 is alive with disease after treatment with azacytidine (9 courses) (Table 3).

**TABLE 3:** Clinical outcome.

\Patients ID	MG2	MG5i	MG4i	MG5	MG6	MG6i	MG7	MG8
Induction treatment	3 + 7	Dead before treatment	Dead before treatment	3−+7	Dead before treatment	Azax9	3−+7	3−+7
Outcome	DDI (d10)	—	—	DDI (d30)	—	Stable disease	RES	DDI (d15)
OS days	40	30	30	60	30	Alive with disease	90	45

DDI, death during induction.

RES, Resistance.

3 + 7, daunorubicin plus cytarabine conventional chemotherapy regimen.

Overall, this scenario is representative of the major current limitation in Kenyan hospitals, meaning the delay in diagnosis and treatment initiation when AML patients are often septic and in very poor clinical conditions.

## Discussion

This study looked at the cytogenetic and molecular abnormalities among AML patients presenting to the hemato-oncology unit of Kenyatta National Hospital. There was no reportable data on the karyotype status of the patients as no cells were cultured from peripheral blood. Increased transit time to the laboratory was the most likely cause for this failure, confirming the lack of feasibility for metaphase cytogenetics in this setting. Of note, despite cytogenetics being obviously relevant for AML prognostication, NGS analysis allowed us to identify lesions that could assign patients to the high risk ELN group independently from karyotyping. We sought to combine WES and targeted DNA sequencing to ensure the highest sensitivity and specificity for detecting mutations associated to myeloid malignancies but still retaining the capability of identify a broader spectrum of mutations by WES.

The study demonstrated, in fact, that patients with AML in KNH do have deleterious mutations that are well-known to be associated with AML pathogenesis. In this regard, although the limited sample size didn’t allow a proper statistical evaluation and cannot be extended to all African cases, the mutational spectrum seemed not significantly different from what has been reported in Western Countries series. Nonetheless, consistent with the overall poor clinical outcome, genetic lesions associated with unfavorable outcome seemed quite common. Three out of eight patients were classified as high risk according to the ELN score for the presence of *RUNX1* (N = 1 patient) and *TP53* (N = 2) mutations. Four were recorded as standard risk, and 1 as favorable (*PML/RARA*+) (Döhner et al., 2017). If also considering *WT1* as adverse risk factor (Virappane et al., 2008; Renneville et al., 2009; Hou et al., 2010), 2 additional patients could be regarded as high risk. Finally, we observed a significantly high occurrence of SNVs affecting *TP53* (overall 5/8 patients) and *ASXL1* (5/8 patients). Even if the specific SNVs were not yet associated with clinical relevance, they cannot be excluded as having potential deleterious role. Similarly, genetic lesions potentially affecting epigenetic regulation (one of the main mechanisms of myeloid malignancies transformation) were common, even if the specific SNVs are not currently associated with a clinical phenotype. Seven out of eight patients presented with *TET2* SNVs, while *DNMT3A* was affected in one out of eight patients.

Overall, the study population seemed to reflect a slightly different scenario from what is commonly observed in AML series. Patients were younger (median age around 30 years vs 68) and the overall treatment response quite poor. Despite younger mean age (43 excluding children), however, only two patients aged below 18 were studied and therefore the series have to be regarded as referring to adults. Pediatric cases definitely need further investigation. This is in line, however, with what was observed in a large series of patients treated at Kenyatta National Hospital and Nairobi Hospital (N. O. Abinya, manuscript in preparation).

This is the first study exploring in depth, the molecular features of Kenyan AML patients, while very few are available on African AML in general. Kappala et al. in South Africa using a microarray-based assessment of molecular variables on AML patients noted that 5.7% had inv (6)/t (16; 16) (p13; q22), 11.3% had t (8; 21) (q22; q22), 3.8% had t (15; 17) (q24; q21), 1.9% had double mutant *CEBPA*, 9.4% had *NPM1*-ABD mutations and 18.9% had a high expression of *EVI1*. *NPM1* mutations were reported at a lower frequency whereas *EVI1* over expression occurred at a higher frequency compared to world data, which would indicate age and racial differences (Kappala et al., 2017). Lower prevalence of *NPM1* and *FLT3*-ITD mutations compared to world data were documented by Marshall et al. looking at a South African cohort with *de novo* AML (Marshall et al., 2014). Awad et al. investigated the prevalence of *FLT3*-ITD mutations of 346 patients with AML in Egypt. 9.2% had t (8; 21), 2.3% had inv (16) and <1% had both t (9; 11) and inv (3) (102). *FLT3*-ITD mutations were present in 18.5% of the total population mirroring the lower frequency of *FLT3* mutation in African studies (Adnan-Awad et al., 2017). Shamaa et al. in Egypt noted a frequency of 34.6 and 28.8% in *FLT3*-ITD and *NPM1* mutations respectively in a cohort of AML patients with a normal karyotype, like western studies. *DNMT3A* and *IDH1*-R132 are frequently mutated in AML patients in Egypt whereas *TET2* overexpression is not a frequent finding (Hamed et al., 2015; Salem et al., 2017; El Gammal et al., 2019). The cytogenetic and molecular patterns of acute myeloid leukemia patients in Africa are difficult to elucidate and compare due to lack of large-scale studies and differing study designs. In India, a country with similar demographics, a large scale analysis of the cytogenetic profile of patients with *de novo* acute myeloid leukemia showed that 15% had t (8; 21), 9% had t (15; 17), 8% had 8+, 6% had −7/del 7q, 5% had *KMT2A* rearrangements, 4.4% had inv (16)/t (16/16), 3% had −5/del 5q, 2% has −17/abn17p and 1.5% had inv (3) in order of decreasing frequency (Amare et al., 2016). Other studies from India show that *FLT3*-ITD, *CEBPA* and *NPM1* mutations occur in 22.3, 8.3 and 8% of patients respectively, a frequency that’s lower than that reported in western data (Ahmad et al., 2012; Khera et al., 2017).

In our study, we could associate targeted DNA sequencing and WES. The concordance between the two was remarkably high, as expected based on the relative specificity and the use of similar chemistry. Only one gene, *STAG2*, turned out to be mutated at targeted sequencing but not at WES in 2 cases. Not being possible to apply a third independent method, we could only speculate that, considering the high specificity of Illumina myeloid panel and the possible lack of sensitivity of WES, it’s indeed more likely that exon capturing didn’t cover the regions of *STAG2* affected by mutations.

On the other hand, WES allowed to detect many SNVs associated with associated comorbidities (e.g., hypercholesterolemia, data not presented). In one case, WES added significant information concerning the molecular pathogenesis of leukemic cells; SNVs potentially associated with cellular transformation as well as drug resistance were, in fact, detected (MG_2). The affected genes included *AURKA*, *ABCB1* and *CBR3* (the two latter associated with drug resistance), *XRCC3, FGFR4, KLC1*, and *NAT2*. Aurora Kinase A (AURKA) has been documented to have some oncogenic activity in the microenvironment milieu of leukemic cells, necessitating search for small molecule inhibitors with anti-leukemic activity (Wang et al., 2020; Du et al., 2021). While MG_2 died during induction he had mutations detected in the *ABCB1* and *CBR3* gene. Evidence have shown that higher expression of *ABCB1* gene (a member of the ATP binding cassette transporters family) results in an increased efflux of chemotherapeutic agents, resulting in drug resistance (Thorn et al., 2011; Shaffer et al., 2012), whereas genetic polymorphisms of *CBR3* (anthracycline metabolizing enzyme) results in differing pharmacokinetics that influence treatment efficacy (Bains et al., 2010). In addition to the aforementioned, mutations in FGFR4 which is a tyrosine kinase receptor, *XRCC3*, *KLC1* and *NAT2* were detected in the same patient. Dysregulated activation of this TKR has been reported to be a significant oncogenic pathway in various solid tumors (Helsten et al., 2016; Liu et al., 2020). Genotypic variants of the DNA repair gene, X-ray cross complementary group 3 (*XRCC3*) have been associated with a significantly higher cancer risk including AML (Bănescu et al., 2013), whereas polymorphisms of *NAT2* an acetylator with epigenetic influences have been described as a modifier of tumorigenesis in various solid tumors (; Zhu et al., 2021). In addition, WES allowed identification of a pathogenic *TERC* mutation in a patient previously affected by AA (Young, 2018; Brzeźniakiewicz-Janus et al., 2020). In this contest, the evolution to AML was accompanied by the acquisition of *RUNX1* mutations.

The present study carries some limitations. First, in this exploratory study, the patients’ population was small and limited to one center, and proper statistical evaluations couldn’t be performed. Second, the paucity of available material didn’t allow to match NGS studies with other techniques exploring both gene mutations (as validation) or protein expression. Indeed, the two applied system validated each other somehow, but further studies are warranted. Finally, despite filtering the analyses for African ethnicity, we cannot exclude that some population-specific genetic lesions could not be captured; in fact, Africans are still underrepresented in genetic databases.

In conclusion, we provided the first study on NGS molecular profiling on Kenyan AML patients, highlighting current limitations of AML diagnostics and treatment in this setting and confirming the relevance of this approach in AML characterization.

## Data Availability

The original contributions presented in the study are included in the article/Supplementary Material, further inquiries can be directed to the corresponding author.
